# Control of *Salmonella* in Chicken Meat by a Phage Cocktail in Combination with Propionic Acid and Modified Atmosphere Packaging

**DOI:** 10.3390/foods12224181

**Published:** 2023-11-20

**Authors:** Wattana Pelyuntha, Kitiya Vongkamjan

**Affiliations:** Department of Biotechnology, Faculty of Agro-Industry, Kasetsart University, Bangkok 10900, Thailand; wpelyuntha@gmail.com

**Keywords:** bacteriophages, biocontrol, food package, food additive, organic acid, shelf-life

## Abstract

*Salmonella* contamination in poultry meat is an important food safety issue as this pathogen can lead to serious illness and economic losses worldwide. In poultry meat processing, a variety of strong bacteriostatic agents has been introduced for controlling *Salmonella* including bacteriophages (phages), organic acids, and modified atmosphere packaging (MAP). In our study, two selected phages including vB_SenM_P7 and vB_SenP_P32 were used in combination with propionic acid (PA) and MAP for controlling *Salmonella* of multiple serovars on chicken meat under storage at 4 °C. The two phages showed strong lytic activity against over 72 serovars of *Salmonella* tested (25.0 to 80.6%). Phages, vB_SenM_P7 and vB_SenP_P32 showed 40% and 60% survival rates, respectively, after the exposure to temperatures up to 70 °C. Both phages remained active, with nearly 100% survival at a wide range of pH (2 to 12) and 15% NaCl (*w*/*v*). The available chlorine up to 0.3% (*v*/*v*) led to a phage survival rate of 80–100%. A combination of *Salmonella* phage cocktail and 0.5% PA could reduce *Salmonella* counts in vitro by 4 log CFU/mL on day 3 whereas a phage cocktail and 0.25% PA showed a 4-log reduction on day 5 during storage at 4 °C. For the phage treatment alone, a 0.3-log reduction of *Salmonella* was observed on day 1 of storage at 4 °C. In the chicken meat model, treatment by a phage cocktail and PA at both concentrations in MAP conditions resulted in a complete reduction of *Salmonella* cells (4–5 log unit/g) on day 2 of storage whereas each single treatment under MAP conditions showed a complete cell reduction on day 4. For the meat sensory evaluation, chicken meat treated with a phage cocktail-PA (0.5%) in MAP condition showed the highest preference scores, suggesting highly acceptability and satisfactory. These findings suggest that a combined treatment using a phage cocktail and PA in MAP conditions effectively control *Salmonella* in poultry meat during storage at low temperature to improve the quality and safety of food.

## 1. Introduction

Poultry meat is often linked with contamination issues by *Salmonella* spp., which inhabits the gastrointestinal tract of live poultry. Contamination in meat can occur during slaughtering and processing [1]. Consumption of food contaminated with *Salmonella* leads to illness. Infectious disease with *Salmonella*, called salmonellosis, is among the most common foodborne diseases reported worldwide. In 2021, salmonellosis affected 60,050 people in EU member states. With most salmonellosis outbreaks caused by *S.* Enteritidis (54.6%), poultry meat is still major source of infection [2]. *S.* Typhimurium was the second most common serovar infecting humans in the EU (11.7%), North America, Australia and New Zealand, and other countries whereas *S.* Infantis, *S.* Hadar, and *S.* Virchow have been listed as the most frequently found serovar [2,3,4]. Overall, salmonellosis causes significant economic losses, with EPSA estimating the total economic burden of human salmonellosis at EUR 3 billion per year [5]. According to the Commission Regulation (EU) No 200/2010 Implementing Regulation (EC) No 2160/2003 of the European Parliament and of the Council, as regards a target for the reduction of the prevalence of *Salmonella* serovars in adult breeding flocks of *Gallus gallus*, 5 serovars as described above were prioritized by EU member states for control of poultry and poultry products entry [6].

During processing at slaughterhouses and processing plants, meat can become contaminated by microflora presented in processing areas and equipment because of unhygienic management. Several methods have been established including chemical methods (organic acids, chloride, phosphate) [7,8,9], physical methods (X-ray, steam, radiation, UV, etc.) [10,11], and natural and synthetic antimicrobials (ionic antimicrobials and bacteriophages) [10,12,13,14]. However, each of these methods alone might not be sufficient to eliminate contaminated microflora that are present on foods or food surfaces. Therefore, the combined methods are of interest for the investigation of a synergistic effect in controlling *Salmonella* populations.

Bacteriophages or phages are the bacteria-killing viruses that have been accepted by the United States Food and Drug Administration (US FDA) as being generally recognized as safe (GRAS) for use as an antibacterial food additive in poultry meat products [15,16]. Phages are extensively studied by researchers due to their strong bactericidal effect against specific bacteria without dangerous impacts on human and animal health. Phages can be applied to reduce *Salmonella* in live poultry at the beginning of cultivation on farms and in poultry meat in processing plants [17,18]. Phage cocktails can expand their lytic ability covering several serovars and prevents bacterial co-infection [19,20]. Similar to phages, propionic acid (PA) has also been approved as GRAS by the US FDA and is widely used as a food additive in several human and animal foods, cosmetics, and pharmaceuticals [21]. PA penetrates microbial cells and decreases the internal pH level—preventing the growth of bacteria and finally killing them [22]. PA is also an alternative to antibiotics used in poultry diets for reducing avian pathogens and diseases [23]. For product shelf-life extension, modified atmosphere packaging (MAP) is commonly used in the poultry industry. MAP has been shown to prevent and control *Salmonella*, other foodborne pathogens, and spoilage micro-organisms in food products [24]. Modified gases within packaging can inhibit bacterial growth (CO_2_), anaerobic growth (O_2_), and prevent lipid oxidation of meat (N_2_) [25].

The current study aimed to evaluate the synergistic effects of a phage cocktail, PA, and MAP in reducing *Salmonella* counts in artificially contaminated chicken meat during storage at low temperature. The physical changes through color change and the meat sensory evaluation after it was treated with the combined treatments were also investigated. Results obtained in the current study will be useful for the poultry industry to consider using the combined treatments as an alternative approach to improve the safety of foods.

## 2. Materials and Methods

### 2.1. Salmonella Phages, Phage Lysate and Phage Cocktail Preparation

Two *Salmonella* phages including vB_SenM_P7 and vB_SenP_P32 were included in this study. Phages vB_SenM_P7 and vB_SenP_P32 were previously isolated from wastewater of animal farm and wastewater treatment station using *S.* Agona H2-016 and *S.* Enteritidis S5-371 as the natural hosts, respectively. *Salmonella* phage stocks were used to prepare 10-fold serial dilutions in salt magnesium (SM) buffer for further overlay preparation on the host lawns following the protocols of Pelyuntha & Vongkamjan (2022) [17]. The overlay was harvested using 5 mL of SM buffer stirred in a horizontal shaker at room temperature for 1–2 h, followed by centrifugation at 6000 rpm for 15 min at 4 °C. The supernatant was filtrated through 0.20 µm syringe filters and phage lysates were kept at 4 °C. The titer of each phage was counted by the forming plaques present on plates of desired dilutions. The phage cocktail was prepared using the same concentration (8 log PFU/mL) of both phages at a ratio of 1:1. Phage titer was also determined by counting plaques as previously described by Pelyuntha et al. (2021) [20].

### 2.2. Salmonella Strains and Phage Host Range Determination

The host range of phages vB_SenM_P7 and vB_SenP_P32 was determined by spotting 10 µL of phage lysate on lawn cultures of each *Salmonella* serovar (72 serovars). These serovars were obtained from different sources that linked to the food production system including foods, broiler farms, broiler slaughterhouses and processing plants, and human sources [20,26]. The overlay lawn was observed for the formation of the clear plaques after growth at 37 °C for 18 to 24 h. Host range determination was performed in triplicate [27].

### 2.3. Phage Stability Tests

#### 2.3.1. Effect of Temperature on *Salmonella* Phage Titer

The effect of different temperatures on phage stability was investigated using a modified method from Ateba & Akindolire (2019) [28]. Each phage suspension (8 log PFU/mL) was incubated at 25, 37, 45, 50, 55, 60, 65, 80, and 100 °C for 1 h. Each treatment was dropped on the double overlay agar plate containing the suspension of each bacterial host. A phage lysate at 4 °C was included as a control. Titer of the phages was monitored following the method previously described.

#### 2.3.2. Effect of pH on *Salmonella* Phage Titer

The pH of the phage suspension (8 log PFU/mL) was adjusted with 1 M HCl or 1 M NaOH to obtain the pH values ranging from 2 to 12. Each treatment was left at 25 °C for 24 h. Titer of the phages was monitored following the method previously described.

#### 2.3.3. Effect of Salinity on *Salmonella* Phage Titer

The suspension of phage (8 log PFU/mL) was blended with NaCl solution to obtain the final concentrations ranging 0.5 to 20.0% (*w*/*v*) and incubated at 25 °C for 24 h. A phage lysate without NaCl treatment was included as a control. Titer of the phages was monitored following the method previously described.

#### 2.3.4. Effect of Free Available Chlorine on *Salmonella* Phage Titer

The suspension of phage (8 log PFU/mL) was blended with free available chorine solution at 0.1, 0.25, 0.5, 1, 2.5, 5% and incubated for 24 h at 25 °C. A phage lysate without chorine treatment was included as a control. Titer of the phages was monitored following the method previously described.

### 2.4. Minimum Inhibition Concentration of Propionic Acid

The broth micro-dilution method was performed to determine the MIC value of PA. Filter-sterile PA was diluted in 96-well microtiter plates containing TSB broth to obtain the final PA concentrations ranging from 0.1 to 1% (*v*/*v*). The inoculum of mixed serovars of *Salmonella* suspension (10 µL) containing 4 log CFU/mL was added to the wells. The fresh TSB broth well was used as the control (no *Salmonella* added), and the inoculum viability (no PA) was used as a positive control. All microplates were incubated at 37 °C for 24 h. MIC values were defined as the lowest concentration of PA that had no visible growth in observed wells [29].

### 2.5. Effect of a Phage Cocktail and Propionic Acid on Salmonella Reduction In Vitro

An overnight culture of each *Salmonella* (*S.* Enteritidis S5-370, *S.* Hadar PPI-013, *S.* Infantis S5-506, *S.* Typhimurium S5-371, and *S.* Virchow H2-117) was mixed and resuspended in TSB and diluted to obtain a final concentration of 4 log CFU/mL. Phage cocktail stock was diluted with TSB to achieve a final phage concentration of 7 log PFU/mL. A 20 mL suspension of *Salmonella* and 20 mL of a phage cocktail or phage cocktail with PA (final concentration at 0.25% and 0.5% *v*/*v*) were mixed at a ratio of 1:1 by volume and incubated at 4 °C. Only mixed culture was served as a control. The number of viable cells from each treatment and control was enumerated at day 0, 1, 2, 3, 4 and 5 by a spread plate on TSA [20]. If the result of viable cell count was at an undetectable level (ND), the presence and absence of any remaining *Salmonella* in the culture broth (25 mL) were also confirmed by the modified ISO 6579: 2017 according to the protocol provided by Biomérieux company and re-streaked on Xylose Lysine Deoxycholate (XLD) agar.

### 2.6. Treatment of Chicken Meat with a Phage Cocktail and PA

Chicken breast was purchased from a supermarket and stored at 4 °C prior to analysis. The chicken breast was cut into a piece of approximately 100 g. To decontaminate the microflora, chicken meat was soaked in 50 ppm of available chlorine solution for 5 min and subsequently washed with sterile distilled water for 5 min three times to remove any available chlorine residue. Chicken meat was soaked in mixed *Salmonella* suspension (*S.* Enteritidis, *S.* Hadar, *S.* Infantis, *S.* Typhimurium, and *S.* Virchow; 4 log CFU/g) for 10 min to allow bacterial attachment. Chicken meat was transferred into linear low-density polyethylene (LLDPE) bags. Then, 1 mL of a phage cocktail, 0.25% (*v*/*v*) PA, 0.5% (*v*/*v*) PA, a phage cocktail with 0.25% (*v*/*v*) PA, a phage cocktail with 0.5% (*v*/*v*) PA, or PBS (control) was added to each bag. All bags were filled with N_2_ at a sample/gas volume ratio of 1:4 (*w*/*v*) connected to an N_2_ cylinder and heat-sealed (tecnovac^®^ Tecnova, Grassobbio BG, Italy). All samples were kept at 4 °C and were examined for microbiological changes (day 0 to 5), physical change (day 0, 3, and 5), and meat sensory evaluation (day 0 and 5).

### 2.7. Monitoring of Salmonella Reduction and Phage Titers in Chicken Meat during Storage

Chicken meat samples were collected on day 0, 1, 2, 3, 4, and 5. Each chicken breast treated was aseptically cut into a piece (approximately 25 g) and mixed with 225 mL of buffered peptone water (BPW) in sterile stomacher bag. The solution in bag was homogenized using stomacher machine with a speed of 220 rpm for 2 min. The mixture was diluted with the same buffer to obtain the appropriate dilution that was spread on XLD agar plates [17]. All plates were incubated at 37 °C for 18 h. The formation of colonies with black centers was observed and recoded. All tests were run in triplicate.

To determine the amount of *Salmonella* phages, the homogenized solution was collected and centrifuged at 6000 rpm for 15 min to settle debris. The supernatant was filtered through 0.20 µm syringe filters and serially 10-fold diluted to obtain the appropriate concentrations. Titer of the phages was monitored following the method previously described.

### 2.8. Evaluation of the Color Change in Chicken Meat

The color change of the chicken meat during storage was evaluated as described by Kim et al. (2014) [30]. Each sample was cut into pieces 2 cm in height and measured with a colorimeter (ColorFlex^®^ EZ, HunterLAB, Hunter Associates Laboratory, Inc., Reston, VA, USA). Lightness (L*) and yellowness (b*) were evaluated. The color was measured five times per sample. Whiteness was calculated using the following equation: Whiteness = L* − 3b*

### 2.9. Sensory Preference Evaluation in Chicken Meat

A total of 50 panelists from the Faculty of Agro-Industry, Prince of Songkla University were included for the evaluation of the overall preference of the meat products. Chicken meat was cut into a small piece (2 cm × 3 cm × 2 cm) and individually kept in a plastic bag. Sample codes with three-digit numbers were presented in random order to avoid carryover effects. Appearance, color, odor, texture, juiciness, and overall liking were the attributes evaluated by each panelist (Table 1).

Liking scores were given for appearance, color, odor, texture, juiciness, and overall liking of samples using a nine-point hedonic scale (1 = dislike extremely, 2 = dislike very much, 3 = dislike moderately, 4 = dislike slightly, 5 = neither like or dislike, 6 = like slightly, 7 = like moderately, 8 = like very much, and 9 = like extremely). Chicken meat treated with MAP alone (control), MAP with a phage cocktail-0.25% PA, and MAP with a phage cocktail-0.5% PA were selected for evaluation based on the 100% reduction of *Salmonella* count both in vitro and in chicken meat. A meat sensory preference evaluation was carried out at day 0 and 5 of meat storage.

### 2.10. Statistical Analysis

Statistical analysis was performed using SPSS (Version 22.0) of Windows statistics software (SPSS Inc., Chicago, IL, USA). The data were subjected to one-way analysis of variance followed by Tukey’s range test. A significant difference between control and treatments was calculated using the independent-samples *t*-test. A difference was also considered statistically significant at the *p*-value < 0.05.

## 3. Results

### 3.1. Phage Host Range Determination

**Figure 1:**
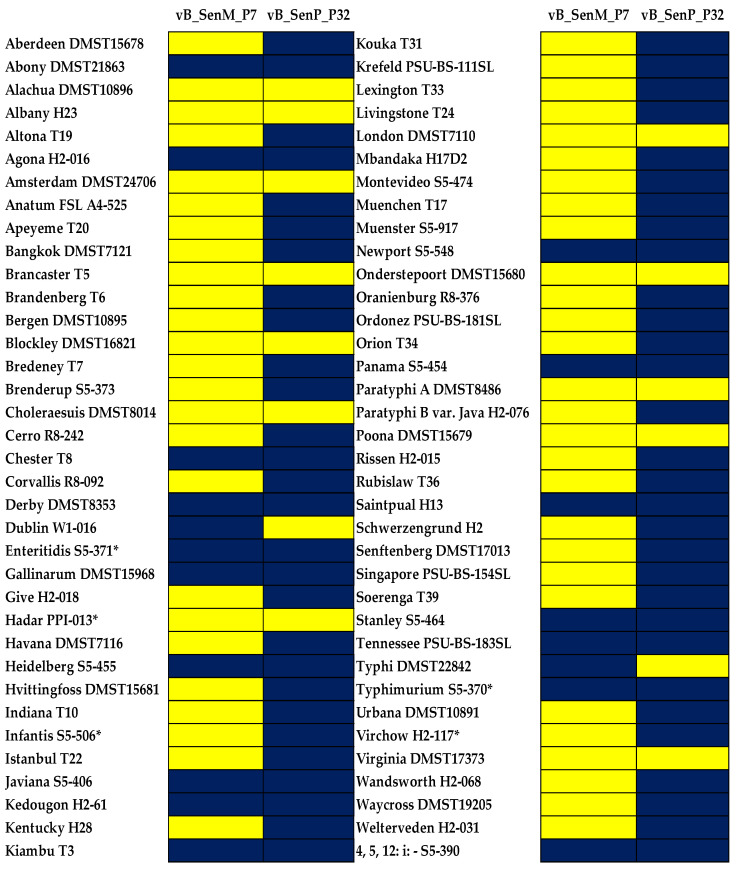
Heatmap representation of the lytic activity of *Salmonella* phages vB_SenM_P7 and vB_SenP_P32 on different *Salmonella* serovars. Serovars with the *asterisk* (*) indicates those that particularly concern the EU. Blue indicates lysis (+). Yellow indicates non-lysis (−).

As shown in Figure 1, two selected phages showed different lysis activity. Phage vB_SenM_P7 showed lower lytic ability against *Salmonella*, presenting 25.0% (lysed 18 serovars) whereas phage vB_SenP_P32 showed the lytic ability as high as 80.6% (lysed 58 serovars). Both phages had strong lytic ability against the two most common serovars in outbreaks worldwide, including *S.* Enteritidis and *S.* Typhimurium. In addition, phage vB_SenM_P7 could lyse *S.* Infantis and *S.* Virchow, which represent the most concerning serovars in the EU [6].

### 3.2. Phage Stability

**Figure 2:**
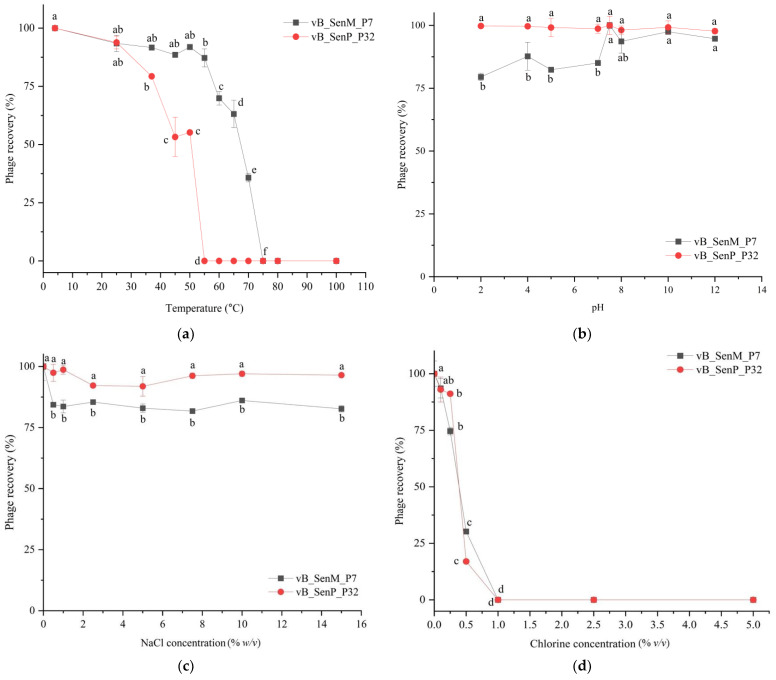
The stability of phages vB_SenM_P7 and vB_SenP_P32 in different conditions; (**a**) temperatures, (**b**) pH (**c**) NaCl concentrations, and (**d**) chlorine concentrations. All values provided as mean ± standard deviation of triplicate. Different lowercase letters indicate the significant difference at the *p*-value of 0.05.

The stability of each phage included in the phage cocktail for further applications was evaluated. Phage vB_SenM_P7 could be recovered up to 35.5% when exposed to a wide range of temperatures between 4 and 70 °C for 1 h (Figure 2a). However, this phage was inactive when exposed to the temperature above 75 °C. Phage vB_SenP_P32 showed 32% survival when exposed to temperatures between 4 and 50 °C (Figure 2a). Phage vB_SenM_P7 remained up to 80% survival when exposed to pH ranging from 2 to 12 while phage vB_SenP_P32 showed high survival rate of nearly 100% when exposed to pH 2 to 12 (Figure 2b). Both phages also showed high survival rates when exposed to NaCl solution up to 15% (Figure 2c). When exposed to chlorine 0.1% and 0.5% for 24 h, phage vB_SenM_P7 showed survival rates of 94% and 30%, respectively, while phage vB_SenP_P32 showed survival rates of 93% and 17%, respectively (Figure 2d). Overall, both phages showed similar survival rates against a wide range of temperatures, pH, salinity, and chlorine concentrations.

### 3.3. Effect of a Phage Cocktail and Propionic Acid on Salmonella Reduction In Vitro

Propionic acid showed the MIC value of 0.25% (*v*/*v*) on *Salmonella* tested. The concentrations of MIC and 2-fold MIC (0.5% *v*/*v*) were used in this study. Reduction of *Salmonella* cells by different treatments was monitored at 1-day intervals for 5 days at 4 °C. A combination of phage cocktail-0.5% PA could completely reduce *Salmonella* count by 4 log CFU/mL on day 3 whereas a combination of phage cocktail-0.25% PA completely reduced *Salmonella* cells later on day 5 (Table 2). Overall, the combination (a phage cocktail with PA) was more effective for controlling *Salmonella* than the single treatment, i.e., phage cocktail or PA alone. On day 5, up to 3-log reduction of *Salmonella* cells was observed in the treatments by a phage cocktail or PA alone when compared to a control (*p* < 0.05).

### 3.4. Reduction of Salmonella in Chicken Meat Treated with a Phage Cocktail and Propionic Acid during Storage at 4 °C in MAP Condition

The significant reduction of *Salmonella* cells between 0.6 to 1.7 log units was observed in all treatments on day 1 of storage (Table 3). Combined treatments of a phage cocktail and PA (0.25% or 0.5%) could completely reduce *Salmonella* cells by 100% (4 log units) on day 2 whereas the PA-treated groups showed a complete reduction of *Salmonella* cells on day 4 of storage. However, a phage cocktail showed greater efficacy in controlling *Salmonella* in MAP condition than PA as indicated by a complete reduction of *Salmonella* cells a day earlier than that observed in PA-treated groups.

In addition, the titers of a phage cocktail remained nearly constant from day 0 to 5 of storage in the treatment with or without PA at 4 °C in MAP condition (Table 4). After 5 days of storage, the titers changed from 7.5 to 7.1 log PFU/g, 6.9 to 6.5 log PFU/g and 6.1 to 6.8 log PFU/g for phages in MAP only, phages with 0.25% PA and phages with 0.5% PA, respectively.

### 3.5. Change of the Meat Color during Storage at 4 °C in MAP Condition

The whiteness was calculated and considered as the typical parameter affecting color changes in chicken meat. The increase of whiteness value in chicken meat was only observed in the MAP group (control), which increased from 9.6 ± 0.0 to 17.2 ± 0.1 on day 5 of storage (Table 5). However, the reduction of whiteness value was observed in all treatments. The whiteness values of chicken meat treated with a phage cocktail only, 0.25% PA, 0.5% PA, a phage cocktail + 0.25% PA and a phage cocktail + 0.5% PA significantly reduced (*p* < 0.05) to 10.7 ± 0.1, 10.3 ± 0.1, 14.5 ± 0.1, 11.8 ± 0.3 and 11.5 ± 0.1, respectively.

### 3.6. Meat Sensory Evaluation

On day 0, the highest preference scores were observed in the treatment with a phage cocktail-0.5% PA. This sample showed a score of appearance, color, odor, juiciness, and overall liking as high as 6.9 ± 0.9, 7.0 ± 1.0, 5.7 ± 1.4, 6.8 ± 1.2, and 6.6 ± 1.1, respectively (Table 6). In MAP conditions after 5 days of storage at 4 °C, scores for the meat appearance, color, and texture preference in the treatment with a phage cocktail-0.5% PA were 7.1 ± 0.9, 7.0 ± 1.0, and 6.8 ± 1.2, respectively. These were higher than the control that showed scores for the meat appearance, color, and texture preference as 6.7 ± 1.2, 5.7 ± 1.4, and 6.7 ± 1.2, respectively. Scores for samples in the treatment with a phage cocktail-0.5% PA were also higher than that in the treatment with a phage cocktail-0.25% PA which showed scores for the meat appearance, color, and texture preference as 6.8 ± 1.3, 6.4 ± 1.3, and 6.6 ± 1.3, respectively. The highest preference for odor was found in the treatment with a phage cocktail-0.25% PA (6.4 + 1.5), followed by a phage cocktail-0.5% PA (6.3 ± 1.2) and the control (5.9 ± 1.5). The highest juiciness preference score was observed in the control (7.1 ± 1.0) whereas the lowest juiciness was found in the treatment with a phage cocktail-0.25% PA (6.6 ± 1.3). From the overall liking, the treatment with a phage cocktail-0.5% PA was still the most accepted from day 0 until day 5 of storage as indicated by a likely score of 6.9 ± 1.0, followed by control (6.5 ± 1.2) and the treatment with a phage cocktail-0.25% PA (6.4 ± 1.3).

## 4. Discussion

*Salmonella* phages isolated from various sources showed differences in the ability to lyse *Salmonella* serovars. Previous studies included 8–29 major serovars of *Salmonella* of concern for food production [17,20,31]. In the present study, 72 major serovars linked to poultry and poultry meat production were included. Although the two selected phages showed differences in the lysis ability against the major serovars included, when they were combined as a phage cocktail, this phage cocktail could cover a broad spectrum of *Salmonella* serovars of concern in the food production from different sources [20,26]. The phage cocktail in the present study could lyse up to four serovars that most concern the EU, including *S.* Enteritidis, *S.* Infantis, *S.* Typhimurium, and *S.* Virchow.

The lytic ability of phages is one of the major criteria when selecting phages for developing a biocontrol targeting foodborne pathogens [32]. Similar studies employed this approach for combining several phages as a phage cocktail to target a wide range of *Salmonella* serovars present in the poultry industry, poultry produce, or other food products [33,34,35,36,37] In addition, previous studies suggest that the use of a phage cocktail can improve the lytic activity against a wide range of multiple serovars. Treatment with a single phage may lead to the development of phage-resistance in bacteria. This is the benefit of using a phage cocktail: if bacteria develop a resistance to one phage, they might be vulnerable to other phages present in a cocktail [18,20].

The two selected phages in the cocktail showed high stability over a wide range of conditions, suggesting the suitability of a phage cocktail to be used on meat products, especially at the post-harvest stage where other harsh processes including some heat treatment, acid-base treatment, chlorine washing, or salt stress may be involved. However, the use of a single approach as in a phage cocktail may still result in the incomplete elimination of *Salmonella* and other bacterial cells in the food matrices [33,38]. In addition, some *Salmonella* cells are resistant and could survive under storage at low temperature [39]. For a phage cocktail treatment in meat products, reduction of *Salmonella* cells between 0.9 and 2.2 out of 6 log units of *S.* Enteritidis and *S.* Typhimurium on chicken breasts were reported after treatment with a cocktail composed of three strong lytic phages (UAB_Phi20, UAB_Phi78, and UAB_Phi87) at 4 °C for 72 h [40]. Another study reported a maximum reduction of 1 log unit on chicken meat after 3 h treated with a phage cocktail (ENT101 and TYM10), while at the end of study, a total of 0.7 log unit reduction was observed when compared to control [41]. Due to the limitation of inhibition by phage cocktail, the remaining bacteria can grow and constantly contaminate until the end of storage.

Organic acids can reduce the growth of several foodborne bacteria present on meat surfaces during storage at low temperature (−16 to 4 °C) [17,42]. PA at 1% and 2% (*v*/*v*) achieved a maximum reduction of *Listeria monocytogenes* in poultry leg after being dipped on the PA solution during storage for 7 days by 2.6 and 2.7 log unit, respectively [43]. In the present study, the single treatment of PA at 0.25% and 0.5% achieved the maximum reduction between 0.3 to 0.5 log units. The combination between a phage cocktail and PA were effective when compared to a single treatment applied in this study. The combined phage cocktail and PA at both concentrations reduced *Salmonella* counts to undetectable levels in vitro and complete elimination of *Salmonella* cells was observed in the meat model after 3 days of storage at 4 °C. Overall, the synergistic effect between the phage and propionic acid treatments could be observed here. Previous studies showed that a phage cocktail combined with lactic acid, paracetic acid, cetylpyridinium chloride, lauric arginate, and peroxyacetic acid could reduce the *Salmonella* counts in chicken breast fillets and freshly trimmed meat [10,44].

The addition of modified gas might play a major role in inhibiting the remaining bacteria present in packaging where a single strategy is used. Consequently, *Salmonella* counts could not be detected on day 3 to 5 of storage. Moreover, the titer of phages presented in each combination was not affected by PA and gas used in the treatment. Phages could survive and remained active until the end of storage. MAP is introduced into the food-processing step in order to improve the product shelf-life by reducing the growth of foodborne and spoilage bacteria in meat, especially *Salmonella*. Djordjević et al. (2018) reported that MAP conditions with 20% O_2_: 50% CO_2_: 30% N_2_ and 20% O_2_: 30% CO_2_: 50% N_2_ reduced *Salmonella* count by more than 2 log CFU/g in minced meat at day 6 of storage [25]. Authors also suggested that MAP containing 50% CO_2_ was more effective than a regular vacuum atmosphere (VA) and MAP with 30% CO_2_. Other strategies are also introduced to combine with MAP such as irradiation, ultraviolet light, essential oils, organic acids, and bacteriophages for increasing the efficacy of MAP to reduce *Salmonella* in foods [10,45,46,47,48].

Regarding the combination of combined phages, propionic acid, and MAP, a synergistic effect was observed in the reduction of *Salmonella* count in chicken meat. Phages can destroy the peptidoglycan present on the bacterial cell wall upon the activity of endolysins. Phage progenies continually reproduced after infecting the same neighbor cells [49]. PA is commonly used as a food preservative and is characterized as an antibacterial compound. PA is superior by inhibiting the growth of Gram-negative and Gram-positive bacteria, as well as yeasts and molds. The mode of action of PA is pH-dependent, which directly affects the redox reduction of NADPH formation, the source of required energy in bacteria [29,50]. MAP is a non-thermal process used for food preservation. O_2_, N_2_, and CO_2_ are typical gases used in MAP packages. O_2_ can be used for inhibiting anaerobic bacteria but does not reduce oxidation, resulting in food spoilage if moisture is present; N_2_ is used to displace oxygen from food packaging. In addition, CO_2_ slows down the growth of different microorganisms. It can freely diffuse into cells, form bicarbonate ions, and release protons. Upon the multiple reactions of CO_2_, the physiological changes of bacteria were observed by alteration of cell membrane function, decrease in the function of enzymes, and change in the physiology of proteins and internal pH [51].

Color of the meat is the one of the most important characteristics for a customer’s purchase decision. The color of chicken meat depends on the level of protein denaturation, storage temperature and pH, and lipid oxidation activity [52,53]. The lower whiteness scores indicate the overall acceptable of the quality of the meat color change. In the present study, the whiteness values of the meat treated with the combined phage cocktail and PA treatments were lower than that in meat from the control group during a 5-day storage. In addition, other sensory preference scores in phage–acid-treated groups were satisfactory without significant difference (*p* > 0.05) when compared to control group, suggesting they were as acceptable as if the meat had never been treated. As pH increases, the negatively charged ions begin to accumulate in higher concentration and cause the repulsion of muscle proteins. When repulsion occurred, the proteins in meat turned from light- to dark-colored. In addition to PA (positively charged ions from the acid) in the meat package, the proteins are not being repulsed, thus the appearance of the meat is pale [53]. Overall, the use of a phage cocktail, PA and MAP for controlling *Salmonella* in chicken meat as a combination did not affect the quality and characteristics of the meat.

## 5. Conclusions

In our study, the combined approach between a phage cocktail and PA provided us with a potential alternative to control *Salmonella* in artificially contaminated poultry meat. These methods did not change the characteristic of the meat, while all examined sensory preferences of the meat were still acceptable. The combination with MAP conditions can be consequently developed to extend the shelf-life of raw chicken meat and related products in large-scale production in the meat industry and to increase the safety of foods. In addition, the whole-genome sequence study of two phages will be in our future plan to ensure the safety of the phages in food production.

## Figures and Tables

**Table 1:** Description of sensory attributes decided upon by the panelists.

Sensory Attributes	Description of Attributes
Appearance	Overall raw chicken meat characteristics
Color	Surface color of raw chicken meat
Odor	Strong to light odor perceived by smelling
Texture	Tight and elastic to loose and friable perceived by hands
Juiciness	Amount of fluid extruded when pressed raw chicken meat between the thumb and forefinger
Overall liking	Overall preference of raw chicken meat

**Table 2:** Monitoring of *Salmonella* populations (log CFU/mL) by different treatments at 4 °C.

Time (Days)	*Salmonella* Count (Log CFU/mL) ^1^					
	Control	Phage Cocktail	0.25% PA	0.5% PA	Phage Cocktail + 0.25% PA	Phage Cocktail + 0.5% PA
0	4.2 ± 0.2 ^a^	4.2 ± 0.1 ^a^	4.3 ± 0.5 ^a^	4.1 ± 0.5 ^a^	4.1 ± 0.1 ^a^	4.3 ± 0.4 ^a^
1	4.1 ± 0.2 ^a^	3.9 ± 0.1 ^a^	3.8 ± 0.2 ^a^	3.9 ± 0.4 ^a^	4.0 ± 0.1 ^a^	4.0 ± 0.3 ^a^
2	4.7 ± 0.1 ^b^	4.1 ± 0.2 ^a^*	3.9 ± 0.2 ^a^*	3.6 ± 0.4 ^a^*	3.7 ± 0.1 ^ab^*	3.3 ± 0.3 ^b^*
3	4.9 ± 0.0 ^b^	4.1 ± 0.2 ^a^*	3.8 ± 0.1 ^a^*	3.9 ± 0.1 ^a^*	3.7 ± 0.1 ^ab^*	ND
4	6.3 ± 0.0 ^c^	3.8 ± 0.1 ^a^*	3.9 ± 0.1 ^a^*	3.6 ± 0.1 ^a^*	3.4 ± 0.4 ^b^*	ND
5	7.0 ± 0.1 ^d^	3.9 ± 0.2 ^a^*	3.8 ± 0.1 ^a^*	3.8 ± 0.1 ^a^*	ND	ND

^1^ All values provided as mean ± SD of triplicate. Different superscript letters indicate a significant difference (*p* < 0.05) of *Salmonella* count in the same treatments. The asterisk (*) indicates the significant difference (*p* < 0.05) between each treatment and the control. ND indicates no *Salmonella* cells detected.

**Table 3:** Monitoring of *Salmonella* populations (log CFU/g) by different treatments in chicken meat during storage at 4 °C in MAP condition.

Time (Days)	*Salmonella* Count (Log CFU/g) ^1^					
	MAP+PBS (Control)	MAP+ Phage Cocktail	MAP + 0.25% PA	MAP + 0.5% PA	MAP + Phage Cocktail + 0.25% PA	MAP + Phage Cocktail + 0.5% PA
0	4.2 ± 0.2 ^a^	4.3 ± 0.0 ^a^	4.4 ± 0.2 ^a^	4.5 ± 0.2 ^a^	4.1 ± 0.0 ^a^	4.0 ± 0.3 ^a^
1	3.6 ± 0.4 ^b^	3.2 ± 0.0 ^b^	3.2 ± 0.2 ^b^	3.0 ± 0.1 ^b^	2.6 ± 0.2 ^b^*	2.3 ± 0.0 ^b^*
2	3.9 ± 0.0 ^a^	2.3 ± 0.2 ^c^*	3.4 ± 0.3 ^b^	2.4 ± 0.1 ^c*^	ND	ND
3	3.6 ± 0.4 ^b^	1.8 ± 0.1 ^d^*	2.7 ± 0.3 ^c^*	2.4 ± 0.0 ^c^*	ND	ND
4	3.8 ± 0.3 ^a^	ND	ND	ND	ND	ND
5	3.9 ± 0.4 ^a^	ND	ND	ND	ND	ND

^1^ All values provided as mean ± SD of triplicate. Different superscript letters in the same treatment of each day are significantly different (*p* < 0.05). The asterisk (*) indicates the significant difference (*p* < 0.05) between each treatment and the control (MAP treatment only) on the same day. “ND” refers to no *Salmonella* count detected.

**Table 4:** Phage titers (log PFU/g) of during storage in various phage treatments.

Time (Days)	Phage Titers (log PFU/g) ^1^		
	MAP + Phage Cocktail	MAP + Phage Cocktail + 0.25% PA	MAP + Phage Cocktail + 0.5% PA
0	7.5 ± 0.1 ^a^	6.9 ± 0.2 ^a^*	6.1 ± 0.9 ^ab^*
1	7.2 ± 0.2 ^a^	7.0 ± 0.3 ^a^	6.2 ± 0.2 ^ab^*
2	7.0 ± 0.3 ^a^	6.7 ± 0.1 ^a^	6.5 ± 0.3 ^a^*
3	6.3 ± 0.3 ^b^	6.7 ± 0.1 ^a^	6.8 ± 0.1 ^a^
4	7.0 ± 0.2 ^a^	7.0 ± 0.3 ^a^	5.7 ± 0.4 ^b^*
5	7.1 ± 0.7 ^a^	6.5 ± 0.4 ^a*^	6.8 ± 0.0 ^a^

^1^ All values provided as mean ± SD of triplicate. Different superscript letters in the same treatment of each day are significantly different (*p* < 0.05). The asterisk (*) indicates the significant difference (*p* < 0.05) between each treatment and the control (MAP + phage cocktail only) on the same day.

**Table 5:** Color change of the chicken meat during storage at 4 ºC in MAP condition.

Treatments	Whiteness ^1^		
	Time (Days)		
	0	3	5
MAP	9.6 ± 0.0 ^a^	15.8 ± 0.5 ^b^	17.2±0.1 ^c^
MAP + Phage cocktail	22.8 ± 0.0 ^a^*	15.8 ± 0.2 ^b^	10.7±0.1 ^c^*
MAP + 0.25% PA	20.7 ± 0.0 ^a^*	14.3±0.6 ^b^*	10.3±0.1 ^c^*
MAP + 0.5% PA	20.1 ± 0.0 ^a^*	18.5±0.1 ^b^*	14.5±0.1 ^c^*
MAP + Phage cocktail+ 0.25% PA	14.9 ± 0.0 ^a^*	12.8±0.4 ^b^*	11.8±0.3 ^c^*
MAP+ Phage cocktail +0.5% PA	23.3 ± 0.2 ^c^*	17.6±0.0 ^a^*	11.5±0.1 ^b^*

^1^ All values provided as mean ± SD of 5 replicate. Different superscript letters in the same treatment of each day are significantly different (*p* < 0.05). The asterisk (*) indicates the significant difference (*p* < 0.05) between each treatment and the control (MAP only) on the same day.

**Table 6:** Meat sensory preference evaluation of the chicken meat kept in linear low-density polyethylene (LLDPE) packaging during storage at 4 °C in MAP condition for 0 and 5 days.

Attributes	Time (Days)	MAP	MAP + Phage Cocktail + 0.25% PA	MAP + Phage Cocktail + 0.5% PA
Appearance	0	6.8 ± 1.5 ^b^	6.3 ± 1.4 ^a^	6.9 ± 0.9 ^b^
	5	6.7 ± 1.2 ^a^	6.8 ± 1.3 ^a^*	7.1 ± 0.9 ^b^*
Color	0	6.7 ± 1.2 ^b^	5.9 ± 1.6 ^a^	7.0 ± 1.0 ^c^
	5	5.7 ± 1.4 ^a^*	6.4 ± 1.3 ^b^*	7.0 ± 1.0 ^c^
Odor	0	5.7 ± 1.6 ^b^	5.4 ± 1.6 ^a^	5.7 ± 1.4 ^b^
	5	5.9 ± 1.5 ^a^	6.4 ± 1.5 ^b^*	6.3 ± 1.2 ^b^*
Texture	0	6.9 ± 1.3 ^c^	5.9 ± 1.6 ^a^	6.6 ± 1.3 ^b^
	5	6.7 ± 1.1 ^a^	6.6 ± 1.3 ^a^*	6.8 ± 1.2 ^a^
Juiciness	0	6.7 ± 1.2 ^b^	6.3 ± 1.5 ^a^	6.8 ± 1.2 ^b^
	5	7.1 ± 0.9 ^b^*	6.6 ± 1.3 ^a^	6.9 ± 1.1 ^ab^
Overall liking	0	6.6 ± 1.2 ^b^	5.9 ± 1.4 ^a^	6.6 ± 1.1 ^b^
	5	6.5 ± 1.2 ^a^	6.4 ± 1.3 ^a^*	6.9 ± 1.0 ^b^*

All values provided as mean ± SD. Total of 50 panelists were included for the evaluation of the overall preference of the chicken meat. Different lowercase letters in the same row indicate the significant difference (*p* < 0.05). The asterisk (*) indicates the significant different between day 0 and day 5 of meat preference evaluation of each attribute in the same treatment.

## Data Availability

The data presented in this study are available on request from the corresponding author.
